# Supplementary material for: Actinobacillus succinogenes in Bioelectrochemical Systems: Influence of Electric Potentials and Carbon Fabric Electrodes on Fermentation Performance
Source: Microorganisms. 2025 Jul 23;13(8):1720. doi: 10.3390/microorganisms13081720 (PMC12388281; doi:10.3390/microorganisms13081720)
Supplement: Supplementary file 1 [file microorganisms-13-01720-s001.zip › microorganisms-3696724-supplementary.pdf]

S1 Supporting information

S1.1 Proteome analysis - Sample preparation

The samples used for proteome analysis were removed from the electrode surface at the respective time points, centrifuged for 10 min at 4 °C and 4000 × g and then flash-frozen in liquid nitrogen without the supernatant. The cell pellets were then lyophilized and stored at -80 °C until further processing. Prior to cell disruption, the pellets thawed on ice. Subsequently, 40 mg of the lyophilized pellets were resuspended in 250 µl of a solution of 8 M urea in 50 mM ammonium bicarbonate. Cell disruption was performed by sonification on ice at a frequency of 20 kHz for a total of 3 min in cycles of 1 s sonification and 2 s pause (550 Sonic Dismembrator Probe, Fisher Scientific, Hampton, NH). The sample was then centrifuged at 8000 × g at 4 °C for 10 min. The supernatant was transferred to a low-binding microcentrifuge tube and centrifuged again at 12000 × g at 4 °C for 20 min. The protein concentration of the resulting supernatant was determined by BCA assay (Pierce™ BCA Protein Assay Kit, Fisher Scientific, Hampton, NH). A protein solution with a total amount of 100 µg protein was then mixed with 20 mM dithiothreitol (DTT) in 50 mM ammonium bicarbonate and incubated for one hour. Then 5 µl of a 475 mM iodoacetamide solution in 200 mM ammonium bicarbonate was added and the sample was incubated at room temperature in the dark. In the next step, the solution was diluted to reduce the urea concentration to 1.5 M to avoid enzyme inhibition. For enzymatic digestion, 5 µg mass spectrometry-grade trypsin was added per sample and the samples were incubated overnight at 37 °C (Trypsin Gold, Promega, Madison, WI). After digestion, detergents and impurities were removed using C18 spin columns (Pierce™ Peptide Desalting Spin Columns, Fisher Scientific, Hampton, NH). The eluted peptide fraction was evaporated and then resuspended in 3% acetonitrile and 0.1% formic acid for LC-MS/MS analysis. After resuspension, the quality of the peptide preparation was checked by measuring the absorbance at 205 nm.

S1.2 Mass Spectrometry Analysis - LC-MS/MS

The proteome analysis was performed by mass spectrometry analysis with liquid chromatography by the Analytical Resources Core of Colorado State University (USA). Reverse phase chromatography was performed using water with 0.1% formic acid (A) and 80% acetonitrile with 0.1% formic acid (B). For each sample, 1 µg of peptides was purified and concentrated using an on-line trap column (Thermo Scientific PepMap Neo C18 5 um, 300 um ID x 0.5 cm). Subsequent chromatographic separation was performed on a Vanquish Neo instrument (Thermo Scientific) on a reverse phase nanospray column with integrated silica emitter (ionopticks Aurora Ultimate Gen 3 C18 1.7 um, 75 um ID x 25 cm column, 45 °C) using a 90 min method at a flow rate of 300 nL/min: 1-6% B over 3 min followed by 6-35% B over 70 min, 35-45% B over 5 min ending in 12 min of washing at 500 $nL\cdot min^{-1}$, 99%B. Peptides were eluted directly into the mass spectrometer (Orbitrap Eclipse, Thermo Scientific) equipped with a Nanospray Flex ion source (Thermo Scientific) and spectra were collected over a m/z range of 375–2000 under positive mode ionization. Ions with charge state +2 or higher were accepted for MS/MS

using a dynamic exclusion limit of 1 MS/MS spectra of a given m/z value with an exclusion duration of 60 s. The instrument was operated in FT mode for MS detection (profile; resolution of 240,000) and ion trap mode for MS/MS detection with a normalized HCD collision energy set to 30% and data centroided.

S1.3 Data Processing

Proteome Discoverer (PD) 3.0 was used for data processing (Thermo Scientific). A precursor detector node with S/N=1.5 was used to identify additional precursors within the isolation window of the precursor spectrum when chimeric or mixed spectra is present. Spectra from all samples were searched using the Sequest HT node, setting methionine oxidation as dynamic and cysteine carbamidomethylation as fixed modification [1]. Subsequently, the data were submitted to an intensity-based rescoring process (INFERYS node in PD) using a deep learning algorithm to predict fragment ion intensities [2]. Raw data was interrogated against the FASTA file of the reference proteome for *Actinobacillus succinogenes* (strain ATCC 55618 / DSM 22257 / CCUG 43843 / 130Z) from Uniprot. Additionally, the cRAP proteome was included (The common Repository of Adventitious Proteins (cRAP) contains commonly found contaminant proteins in proteomics experiments) fasta file "cRAP_100518". Sequest HT was searched with a fragment ion mass tolerance of 0.60 Da and a parent ion tolerance of 10 PPM. Peptide spectral matches were validated using the Percolator node [3]. Thresholds were set such that a false discovery rate of ≤1% and protein identification was defined with at least one peptide.

References

[1] R. K. Scopes, "Measurement of protein by spectrophotometry at 205 nm," Anal Biochem, vol. 59, no. 1, pp. 277–282, 1974, doi: 10.1016/0003-2697(74)90034-7.

[2] J. K. Eng, A. L. Mccormack, and J. R. Yates, "An Approach to Correlate Tandem Mass Spectral Data of Peptides with Amino Acid Sequences in a Protein Database," 1994.

[3] D. P. Zolg et al., "INFERYS rescoring: Boosting peptide identifications and scoring confidence of database search results," 2021, doi: 10.1002/rcm.9128.
